# PRIMERS: Polydopamine Radioimmunotherapy with Image-Guided Monitoring and Enhanced Release System

**DOI:** 10.3390/pharmaceutics16111481

**Published:** 2024-11-20

**Authors:** Shahinur Acter, Lindokuhle M. Ngema, Michele Moreau, Debarghya China, Akila Viswanathan, Kai Ding, Yahya E. Choonara, Sayeda Yasmin-Karim, Wilfred Ngwa

**Affiliations:** 1 Department of Radiation Oncology and Molecular Radiation Sciences, Johns Hopkins School of Medicine, Baltimore, MD 21287, USA; lindokuhle.ngema@wits.ac.za (L.M.N.); mmoreau1@jh.edu (M.M.); anv@jhu.edu (A.V.); kding1@jhmi.edu (K.D.); 2 WITS Advanced Drug Delivery Platform Research Unit, Department of Pharmacy & Pharmacology, University of Witwatersrand, 7 York Road, Parktown, Johannesburg 2193, South Africa; yahya.choonara@wits.ac.za; 3 Department of Biomedical Engineering, Johns Hopkins School of Medicine, Baltimore, MD 21287, USA; dchina1@jhmi.edu; 4 Department of Radiation Oncology, Brigham and Women’s Hospital, Dana-Farber Cancer Institute, Boston, MA 02115, USA; syasmin-karim@bwh.harvard.edu

**Keywords:** polydopamine, image-guided drug release, fiducial markers, cancer, combination therapy, mesoporous bowl-shaped nanoparticles

## Abstract

**Background/Objectives:** To overcome the side effects of conventional cancer treatment, multifunctional nanoparticles with image-guidance properties are increasingly desired to obtain enhanced therapeutic efficacy without any toxicity of the treatment. Herein, we introduce the potential of Polydopamine Radioimmunotherapy with Image-guided Monitoring and Enhanced (drug) Release System (PRIMERS) to meet the challenges of currently used cancer therapy. **Methods:** The PDA nanobowls were synthesized using an emulsion-induced interfacial anisotropic assembly method followed by surface modification with high-Z material to obtained the final product PRIMERS. **Results:** The engineered multifunctional nanosystem “PRIMERS” could serve as fiducial markers with the potential for use in combination cancer therapy. By leveraging the advantages of the excellent surface functionalization capability of PDA, the anisotropic nanostructure (PDA nanobowls) has been successfully functionalized with gadolinium, which shows strong MRI contrast signal both in vitro in phantom and in vivo in animals. The results of anti-cancer drug loading and releasing efficiency of these functionalized nanobowls are presented. Moreover, the gadolinium-coated PDA nanobowls demonstrate the capacity for loading immunotherapy drugs (Anti-CD40) with activated release in acidic pH levels characteristic of the tumor microenvironment, with enhanced release following administration of radiation therapy in vitro. **Conclusions:** Overall, the results highlight the potential of this new technology for combining radiotherapy with activated image-guided drug delivery, which offers broad opportunities to overcome current challenges in cancer treatment.

## 1. Introduction

Currently used traditional cancer therapies include surgery, radiotherapy, chemotherapy, and immunotherapy, which have limitations in terms of therapeutic efficacy and toxicities [1,2,3,4]. Therefore, cancer still remains a leading cause of death and morbidity in the world [2,5]. Multifunctional nanoparticles are increasingly desired to obtain enhanced therapeutic efficacy and minimize the side effects of conventional cancer treatment [6,7,8,9,10,11,12]. In particular, combined immunotherapy and radiation therapy along with chemotherapy may potentiate significantly enhanced treatment efficacy, as well as image-guidance capability, which is valuable for guiding treatment and monitoring drug delivery [13,14,15,16,17,18].

Fiducial markers are small objects or medical devices that are placed in or on the body to mark an area, and are currently used to provide image guidance during radiotherapy, providing an ability to accurately track the location and position of the tumors, especially for tumors that may move during the treatment [19,20,21]. In a number of studies, fiducial markers demonstrate the capability to improve the precision of radiation therapy, thus minimizing damage or toxicity to surrounding healthy tissues, which eventually improves the therapeutic efficacy [22,23]. Since the 1990s, fiducial markers have been playing an essential role in image-guided radiotherapy for cancer treatment [22]. Fiducial markers are surgically implanted inside the location of interest prior to radiotherapy [23,24]. To ensure positioning accuracy, fiducial markers are engineered to be implanted in the location of interest and are currently in solid or liquid forms [22]. Organic dyes attached to particles are one of the more commonly used fiducial markers that show contrast in electron microscopy and correlative light and electron microscopy (CLEM) [25]. However, this type of organic dye-based fiducial markers comes with the limitation of photobleaching, as they are not very stable and induce electron beam exposure [26,27]. More recently, an increasing number of studies have investigated injectable fiducial markers based on nanocarriers [19]. However, the development of nanoparticle-based fiducial markers with multifunctional properties has been desired for image-guidance combination therapy to overcome the current limitations in cancer treatment.

Nanoparticles-based fiducial markers are designed to be bio-compatible and intrinsically nonimmunogenic without considering the ability to provide additional functions beyond image guidance, such as for the image-guided drug delivery of immunotherapy or chemotherapy [28]. However, in the development of multifunctional nanoparticles for combination cancer therapy, polydopamine (PDA) has gained enormous research attention in recent years due to its flexibility in designing desired morphology and size along with excellent surface functionalization properties [2,29,30,31]. It is an oxidative polymerized product of monomer dopamine (DA) in a higher pH environment, which is known as a neurotransmitter, and various studies have been conducted exploring the potential of DA in the biomedical fields in particular brain diseases [32,33]. Moreover, PDA is known for its excellent biocompatibility, biodegradability, and colloidal stability in the biological system [34]. In several studies, PDA nanobowls have shown efficacy in combination cancer treatment with chemo- and photothermal therapy [30]. Additionally, the higher cellular uptake efficiency due to their bowl shape and the possibility of loading sufficient amounts of drugs and molecules into the mesopores and cavity make this anisotropic shaped nanoparticle unique [30,35].

In our study, for the first time, we integrate this knowledge combined with facial synthesis and a functionalization approach to develop a multifunctional fiducial marker, Polydopamine Radioimmunotherapy with Image-guided Monitoring and Enhanced (drug) Release System (PRIMERS), for precise drug localization and activated release. This study shows the potential of PRIMERS as fiducial markers, as well as justifying future investigations exploring their therapeutic use to meet current challenges in cancer treatment.

## 2. Materials and Methods

**Figure 1:**
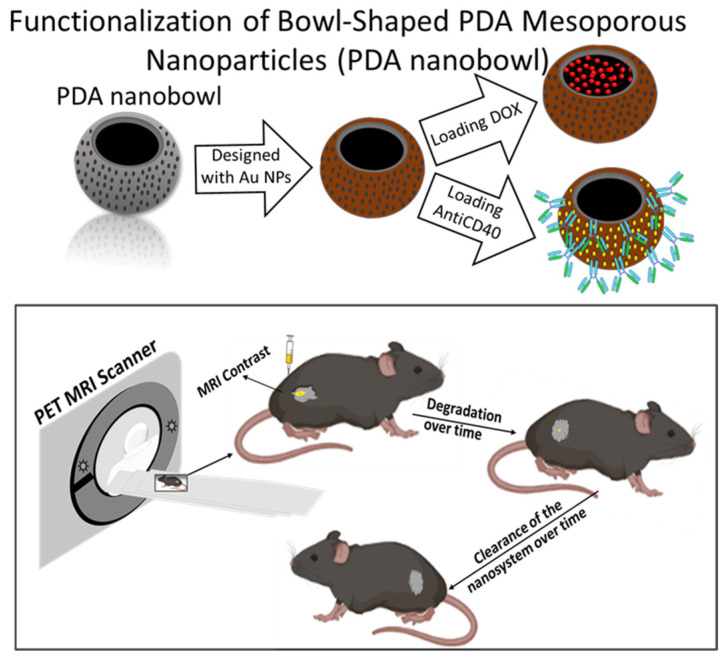
Schematic diagram showing the functionalization of polydopamine bowl-shaped mesoporous nanoparticles (on **top** and on **bottom**) showing MRI contrast of the nanosystem in a mouse model.

The PRIMERS is developed by functionalizing PDA nanobowls via coating them with metal (gadolinium) followed by loading them with anticancer drugs whose release can be activated by an external stimulus. A schematic diagram is shown in Figure 1.

### 2.1. Formation and Characterization of PDA Nanobowls

The PDA nanobowls were designed following an established method known as an emulsion-induced interfacial anisotropic assembly [36]. Briefly, 1.5% (*w*/*v*) dopamine hydrochloride (DA) and 1.0% (*w*/*v*) Pluronic F127 (block copolymer) were dissolved in a water:ethanol (1:1) mixture (with a total volume of 10 mL) followed by the addition (dropwise) of 2.0% (*v*/*v*) 2,4-trimethylbenzene (TMB) under stirring. Ultra-sonication was used for 2 min to form the emulsion system. An alkaline environment was created by adding ammonia (NH_4_OH, 28%) solution (3.75% (*v*/*v*) dropwise into the emulsion system. After 2 h, synthesized particles were centrifuged with a water and ethanol mixture three to four times. The final product was freeze-dried for characterization and future use. The synthesized nanoparticles were characterized by dynamic light scattering (DLS), and phase-analysis light scattering (PALS) analyses were conducted to discern the average hydrodynamic size and zeta potential (surface charge) using a ZetaSizer instrument (NanoZS, Malvern Panalytical, Malvern, UK). Later on, transmission electron microscopy (TEM (Hitachi 7600 TEM operating at 80 kV with an AMT XR80 CCD (8 megapixel)) was used to examine the size and morphology of the particles. Fourier transform infrared (FTIR) spectroscopy (Spectrum 100, PerkinElmer Inc., Waltham, MA, USA) was used for the chemical characterization of the nanoparticles. This was done to confirm the formation of PDA nanobowls. Dried samples were appropriately analyzed at 120 psi pressure in a range of 4000–650 cm^−1^ over 20 scans to obtain relevant spectra.

### 2.2. Coating PDA Nanobowls with Gadolinium and Characterization

To achieve the magnetic resonance imaging (MRI) contrast of PDA nanobowls, a thin layer of gadolinium coating was created by adding gadolinium chloride (III) (from Sigma Aldrich, St. Louis, MO, USA) into the solvent (1:1, water:ethanol) during the nanoparticle synthesis process. Herein, various concentrations of gadolinium chloride solution (40, 100, and 150 µg/mL) were used, which produced PDA bowls with various thicknesses of gadolinium on the surface. After the polymerization time, the nanoparticles were washed five times to remove free gadolinium. The transparent color of the supernatant was the initial indication of the successful washing of the nanoparticles and the complete removal of the unwanted residue. All supernatants were collected for further analysis. The final product was freeze-dried for characterization and future use.

After coating with gadolinium the PDA nanbowls were characterized by dynamic light scattering (DLS), and phase-analysis light scattering (PALS) analyses were conducted to discern the average hydrodynamic size and zeta potential (surface charge), using a ZetaSizer instrument (NanoZS, Malvern Panalytical, Malvern, UK). Later on, transmission electron microscopy (TEM (Hitachi 7600 TEM operating at 80 kV with an AMT XR80 CCD (8 megapixel)) was used to examine the size and morphology of the particles. FTIR spectroscopy (Spectrum 100, PerkinElmer Inc., Waltham, MA, USA) was used to comparatively analyze the chemical integrity and functional group transitions in pristine PDA nano bowls and gadolinium coated PDA nano bowls. This was done to confirm the formation of PDA nano bowls and successful coating with gadolinium. Dried samples were appropriately analyzed at 120 psi pressure in a range of 4000–650 cm^−1^ over 20 scans to obtain relevant spectra.

### 2.3. Loading Doxorubicin (DOX) into Gadolinium-Coated PDA Nanobowls and Release Study of DOX

To evaluate the drug delivery properties of gadolinium-coated PDA nanobowls, the anti-cancer drug doxorubicin (DOX) was loaded into the nanobowls based on the approach of a previous study [30]. For loading DOX, a 1 mg/mL nanobowls (total volume 5 mL) suspension was prepared, and various quantities of DOX (0.5, 1.0, 1.5, 2.0, 2.5, and 3 mg) were dispersed in the nanobowls suspension, and this mixture was stirred for 24 h at room temperature to allow binding to the nanobowls. Afterward, ultrapure water was used to wash the DOX-loaded gadolinium-coated PDA nanobowls by centrifugation three to four times to remove free DOX from the nanosystem. This process ended by freeze-drying the resulting dox-loaded PRIMER for future use. To estimate the drug loading capacity of the PRIMER, all supernatants were collected. Herein, the loading efficiency of DOX was determined by fluorescence spectrophotometry (microplate reader spectraMax) at an excitation wavelength of 480 nm and an emission wavelength of 590 nm compared to a standard curve (Figure S3, Supplementary Document). DOX loading efficiency into the nanosystem was calculated by subtracting the mass of the DOX in the supernatant from the total mass of the DOX in the initial solution divided by the total amount of DOX, and expressing it as a percentage. An in vitro release study of DOX was performed under pH 5.6 conditions, followed by a previous study, where acidic conditions have been found beneficial in releasing DOX [37]. Herein, 1 mL PDA nanobowls/ DOX (1% loading efficacy) were loaded into a dialysis bag (MWCO 3.5 kDa) with 25 mL 1× PBS buffer solution. The pH of the PBS buffer solution was modified to pH 5.6. 1 mL PBS solution was collected at different time intervals, and fluorescence spectrophotometry was used to quantify the amount of released DOX at a wavelength 590 nm. Here, 1 mL fresh PBS was added into the solution at each sampling time point in order to keep the total volume constant.

### 2.4. Loading AntiCD40 in Gadolinium-Coated PDA Nanobowl and Release Study of AntiCD40

To investigate the antiCD40 loading efficiency in the PRIMERS, Alexa fluoro700 anti-human CD40 (Vol. 5 µL, Con. 200 µg/mL) was added in the particles suspension (50 mg/mL nanobowls (total volume 20 mL). The suspension was prepared in 25 mM MES buffer (pH 6) [38]. The loading experiment was conducted at 4 °C by slow shaking overnight. Afterward, antiCD40 loaded PRIMERS were washed several times (four to five times) by centrifugation at 13,000 rpm to remove the free antiCD40. All experimental procedures here were conducted at 4 °C in a sterile environment. All supernatants were collected for further analysis. Fluorescence microscopy (Zeiss LSM880-Airyscan, 07745 Jena, Germany, FAST Super-Resolution) was used to characterize the PRIMERS. In vitro, in a sterile environment, an antiCD40 release study was performed in various solvents with and without irradiation exposure in a petri dish at 4 °C.

### 2.5. MR Imaging

To assess its ability to provide contrast images for treatment guidance and drug delivery monitoring, MRI imaging of the PRIMERS was conducted in vitro and in vivo using a 7T Bruker PET-MR Scanner. For in vitro imaging, the sample was injected in an agar gel phantom. The same concentration and volume of PRIMER components were injected into each phantom. For in vivo experiments, MRI imaging was investigated in *C57BL6* mice bearing cervical subcutaneous tumors. T1 weighted MRI images from the entire tumor region were collected over different time points, and the imaging was done over several weeks. All experiments were performed under anesthesia.

The volumes and contrast of the tumor and nanoparticles were semi-automatically quantified from the acquired MRI datasets using the MITK Workbench version 2023.04. Subsequently, these volumes were visualized with 3D Slicer version 5.2.1. Further graphical visualization and analysis of the volume and contrast for both the tumor and nanoparticles were performed using Python version 3.9.

### 2.6. In Vitro Cytotoxicity Assay

The cell viability of the designed PRIMERS was determined using an MTT cell viability assay. TC-1 cells were first seeded at a density of 2 × 10^4^ cells mL^−1^ in a 96-well plate with a volume of 50 μL in each well and left to adhere for 24 h at 37 °C and 5% CO_2_. The assay was conducted in triplicates with various concentrations of nanobowls that were non-coated as well as gadolinium-coated ones (100 and 500 μg mL^−1^) for 24 h at 37 °C under 5% CO_2_. After the desired incubation time, the absorbance at 550 nm was recorded using a SpectraMax microplate reader. The absorbance values of the control (untreated cells) were set at 100%, and cell proliferation was expressed as a percentage of the control. This study was conducted to investigate the cytotoxicity of the PRIMER components without the drugs or antiCD40 loaded into them.

### 2.7. TC-1 Cell Culture and Tumor Growth in C57BL6 Mice

A cervical cancer cell line, TC-1 (ATCC^®^ CRL 2785, Manassas, VA, USA), was used to grow subcutaneous tumors in *C57BL6* female mice following a previously published protocol [39]. The TC-1 cells were cultured in RPMI 1640 medium supplemented with 2 Mm L-glutamine adjusted to contain 1.5 g/L sodium bicarbonate, 4.5 g/L glucose, 10 mM HEPES, 1.0 mM sodium pyruvate, 0.1 mM non-essential amino acids, and 10% fetal bovine serum in a humidified incubator at 37 °C under 5% CO_2_. *C57BL/6* strain female mice were acquired from Charles River at 6–8 weeks old. They were inoculated subcutaneously with 3 × 10^5^ cells mixed with Matrigel in the lateral flanks of mice and allowed to grow to a palpable tumor size of approximately 3 mm in diameter within ten days before the start of treatment. The formula V = 0.5 × length × (width^2^) was used to determine mice tumor volume. A digital caliper was used to measure the longitude protrusion of the tumor, designated as the tumor length and the latitude projection as the tumor width. All the animal experiments were conducted as per the guidelines and regulations of the Johns Hopkins University Animal Care and Use Committee (ACUC) set under protocol #MO21M281. Mice maintenance in the Johns Hopkins University animal facility was done according to the Institutional Animal Care and Use Committee approved guidelines.

### 2.8. Human Umbilical Vein Endothelial Cells (HUVEC) Cell Culture

Human umbilical vein endothelial cells (HUVEC) were prepared as in the previous study [40]. It is an endothelial cell line that was isolated from the vein of the umbilical cord (ATCC 30-2004, Manassas, VA, USA). HUVEC cells were cultured in F-12K medium supplemented with 0.1 mg/mL heparin, 500 μL of 30 mg/mL endothelial cell growth supplement (ECGS) (fisher scientific, Hampton, NH, USA, BE0016-2) and 10% fetal bovine serum (ATCC 30-2020, Manassas, VA, USA) in a humidified incubator at 37 °C under 5% CO_2_. Cells were starved for 1 h in basal media, which was then replaced with the growth medium, and incubated with various concentrations of PDA nanobowls for 72 h.

## 3. Results and Discussions

### 3.1. Preparation and Characterization of PRIMERS Precursor Polydopamine Bowl-Shaped Mesoporous Nanoparticles (PDA Nanobowls)

**Figure 2:**
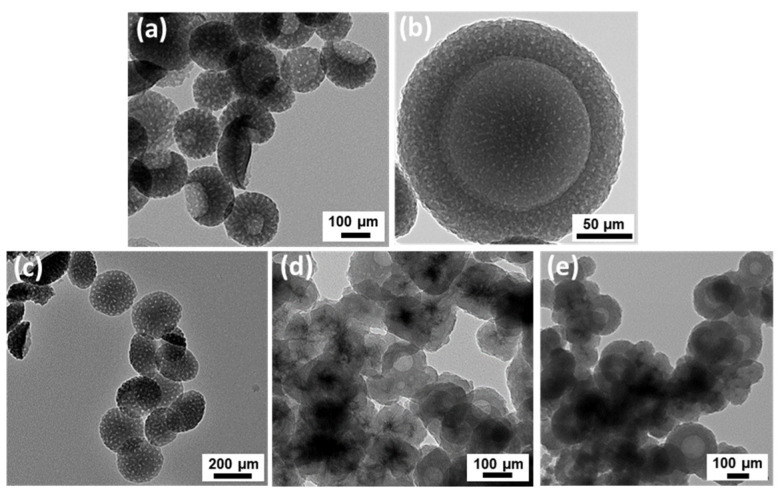
Transmission electron microscope (TEM) image of PDA nanobowls before functionalization (**a**,**b**), with (**b**) being a high-magnified view of the PDA nanobowl (**a**), and (**c**–**e**) showing PDA nanobowls coated with various concentrations of gadolinium (40, 100, and 150 µg/mL, respectively).

The PRIMERS was developed from polydopamine bowl-shaped mesoporous nanoparticles (PDA nanobowls), which serve as a precursor for functionalization and drug loading. In our study, an emulsion-induced interfacial anisotropic assembly method was used to synthesize PDA nanobowls; herein, PDA bowls at around 200 nm were chosen considering their cellular uptake efficiency determined in previous study by Acter and their group. Building on a previous study, the synthesis process of these anisotropic nanobowls is mainly based on the simultaneous formation of Pluronic^®^ F-127/TMB/PDA composite micelles, and the nucleation of PDA and subsequent anisotropic growth of PDA on the surface of the emulsion droplet templates [36]. Previous studies demonstrate that by simply adjusting the concentration of pore swelling agent TMB, surfactant F127, monomer (dopamine), and aqueous ammonia solution, the size and morphology of the PDA nanobowls can be controlled [29]. In Figure 2a, the monodispersity of the nanobowls is observed in a TEM image, where the diameter of the nanobowls was found at around 200 nm with a cavity with an approximate width of 80 nm (Figure 2a). Higher magnification revealed the pore size of the nanobowls, which is estimated to be around 7 nm (Figure 2b)). Additionally, in all the TEM images, as represented in Figure 2a,b), a clear view of the mesochannels is achieved radially from the center to the surface of the bowls, and the center-to-center distance between adjacent mesochannels is estimated to be ≈21 nm, as determined by higher magnification TEM image. A graphical representation of (a) the average particle size and (b) the zeta potential of the synthesized PDA nanobowls is given here. An average size analysis showing the size of the PDA nanobowls is given in Figure S1C. In previous studies, we have demonstrated the size distribution and zeta potential of PDA nanobowls for this formation process using dynamic light scattering (DLS) and phase analysis light scattering (PALS), respectively, with negative zeta potentials of the nanobowls with moderate magnitudes in both water and cell culture medium, suggesting colloidal stability [30,35]. Chemical characterization of the PDA nanobowls was also accomplished by Fourier transform infrared spectroscopy (FTIR), as shown in Figure S1D [35].

### 3.2. Functionalization of PDA Nanobowls

#### 3.2.1. Gadolinium Coating on the PDA Mesoporous Nanobowls

**Figure 3:**
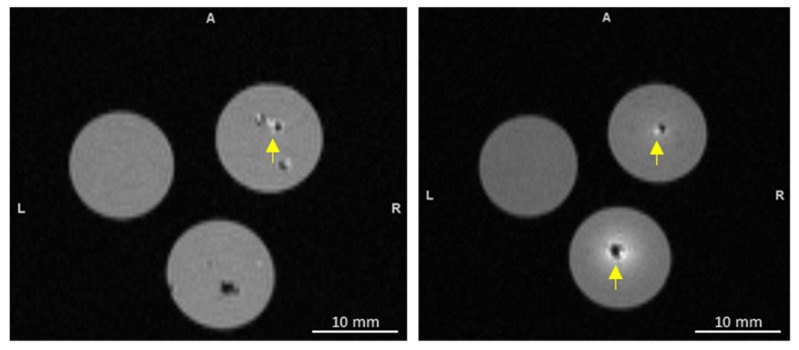
Showing the MRI contrast of gadolinium-coated PDA mesoporous nanobowls in agar gel phantom. Image on the left: agar gel phantom only on the left, PDA nanoparticles in phantom on the bottom, and gadolinium-coated PDA nanobowls (gadolinium (III) chloride (100 µg/mL)) on the top right. MR image on the right: agar gel phantom only on the left, top right and bottom show gadolinium-coated PDA nanobowls with 100 µg/mL and 150 µg/mL of gadolinium (III) chloride, respectively. Herein, the letter A refers to the additive intensity value for an ellipse in a phantom image, L refers to Left and R refers to Right.

As mentioned earlier, the PDA surface is designed with catechol-based functional groups, which have a strong ability to form coordination bonding with metal ions [2]. Taking the advantages of the catechol-based functional groups on PDA, surface-controlled coating with gadolinium was achieved on the PDA mesoporous nanobowls in situ. In a series of experiments, various concentrations of gadolinium chloride were used to coat the surfaces of PDA nanobowls. To confirm the coating of gadolinium, TEM imaging analysis was conducted. The TEM images in Figure 2c to e indicate that with increased concentrations of gadolinium chloride, the thickness of the gadolinium on the PDA surface increased; after the thin coating on the PDA surface, the mesopores can be clearly observed, with a slight increase in the size of the nanobowls (Figure 2c–e). Moreover, the mesopores were filled with gadolinium (Figure 2d,e). FTIR analysis confirms the successful coating of Gd with PDA nanobowls (Figure S1D). Size distribution and zeta potential measurements of Gd-coated PDA nanobowls (various concentrations of Gd) are shown in the supporting documents in Table S1, with graphical representations of the (A) average particle size and (B) zeta potential of the synthesized Gd-coated PDA nanobowls. As demonstrated in Figure S1C, the size of the Gd-coated PDA nanobowls increased with higher concentrations of Gd coating, which further confirms the successful coating of Gd on the PDA surface. In a number of studies, it has been demonstrated that gadolinium-based nanoparticles can provide imaging contrast during magnetic resonance imaging (MRI) and boost the efficacy of radiation therapy. MRI gadolinium is commonly used because of its seven unpaired electrons, which benefit it with one of the most paramagnetically stable metal ions. Gadolinium molecules shorten the spin-lattice relaxation time (T1) of voxels in which they are present, resulting in a brighter signal on T1 weighted images. The magnetic properties of gadolinium or gadolinium-based contrast agents in combination with magnetic resonance imaging (MRI) allow blood vessels, organs, and other non-body tissues to be seen clearly during MR imaging. Therefore, this rare earth metal is commonly applied in diagnosis and image-guidance therapy [41,42]. To investigate the potential of gadolinium-coated PDA mesoporous nanobowls as MRI contrast agents for PRIMERS, a 7-tesla (T) MRI scanner was used. As demonstrated, compared to control 1 (only phantom) and control 2 (phantom injected with PDA nanobowls without gadolinium coating), the samples (PDA nanobowls with gadolinium coating) provide enhanced positive contrast during MRI scanning (Figure 3).

The results show that the phantom images evidently become brighter as the gadolinium concentrations increased, consistent with the expectation that an increased concentration of gadolinium in the nanosystem accelerates the recovery of net magnetization.

**Figure 4:**
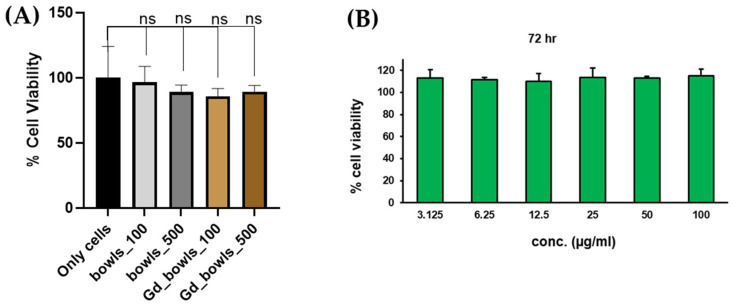
(**A**) Showing the (3-[4,5-dimethylthiazol-2-yl]-2,5 diphenyl tetrazolium bromide) (MTT) assay of polydopamine (PDA) mesoporous nanobowls with and without gadolinium coating in TC-1 cells (concentration of gadolinium 100 µg/mL), *p* value (only cells vs. bowls_100 µg/mL—0.8380; only cells vs. bowls_500 µg/mL—0.4842; only cells vs. Gd_bowls_100 µg/mL—0.3737; Gd_bowls_500 µg/mL—0.4872), (**B**) MTT assay results showing biocompatibility of PDA mesoporous nanobowls on human umbilical vein endothelial cells (HUVEC). Herein, ns refer to non-significant value.

Before investigating the potential use of the gadolinium-coated PDA nanobowls as fiducial markers in vivo, their toxicity needed to be evaluated in vitro. The cell viability of the nanosystem has been investigated in TC-1 cells (Figure 4A) and human umbilical vein endothelial cells (HUVEC) (Figure 4B) using an MTT assay kit, which showed no obvious cytotoxicity with the incubation of gadolinium-coated PDA nanobowls. This was observed even with the concentration of the nanoparticles reaching 500 µg/mL, with over 80% of the cells viable after 24 h of incubation time (Figure 4A). The obtained results suggest the biocompatibility of PDA nanobowls (Figure 4B). Additionally, a cellular internalization study of PDA nanobowls in TC-1 cells was conducted, and the confocal microscopy images in Figure S2 show a significant number of PDA nanobowls were taken up by the TC-1 cells in 24 h. In this study, in vivo experiments were conducted in a mouse model by injecting the gadolinium-coated PDA nanobowls intratumorally.

**Figure 5:**
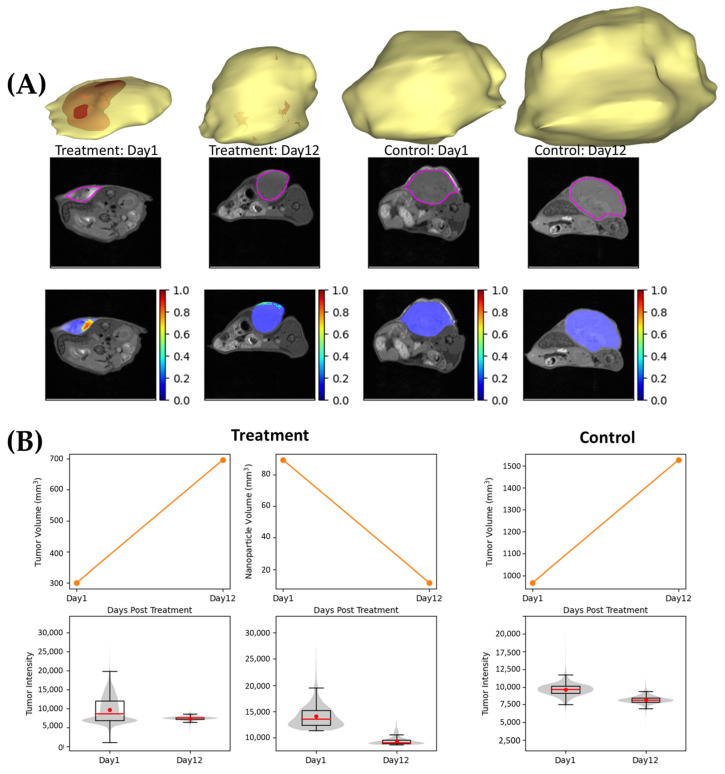
(**A**) MRI images of the tumor showing PDA mesoporous nanobowls coated with 100 µg/mL of gadolinium (PRIMERS) and tumor visualization over time. Treatment means the PRIMERS is administered intratumorally, and control means no PRIMERS administration. (**B**) Showing the quantitative analysis of MRI images; on the left side, the two sets of images are treatment samples from day 1 and day 12. On the right side, the two sets of images are control samples from day 1 and day 12. Herein, treatment means the PRIMERS was intertumorally administered, and control means the PRIMERS was not administered. Herein, the pink box refers to the area of the tumour, where the MRI contrast is expected to observed.

A positive MRI signal contrast was also observed in T1 weighted images of the tumor after the intra-tumoral injection of the gadolinium-coated PDA nanobowls. As observed in Figure 5, strong image contrast is observed at day 1 post-injection of the nanosystem; however, the significant fading of the MRI signal was observed from day 12 until the tumor reached the endpoint (Figure 5). These results confirm the potential use of the gadolinium-coated PDA nanobowls as fiducial markers in vivo, especially for image-guided hypofractionated radiotherapy or radioimmunotherapy, as shown in previous work using one radiotherapy treatment fraction. The system can be further optimized to customize treatment schedules. The results also suggest the clearance of the nanoparticles from the biological system after a certain period of time due to the biodegradation of the nanoparticles. Additionally, the quantitative analysis in Figure 5B further confirms the successful administration and degradation over time of the PRIMERS as the intensity of the tumor and nanoparticles (MRI contrast of the PRIMERS) changes over time with respect to the control. These results highlight the potential use of the gadolinium-coated PDA nanobowls as biodegradable fiducial markers in vivo.

#### 3.2.2. PRIMERS: Loading Anti-Cancer Drug in Gadolinium-Coated PDA Nanobowl and Drug Release Profile In Vitro

**Figure 6:**
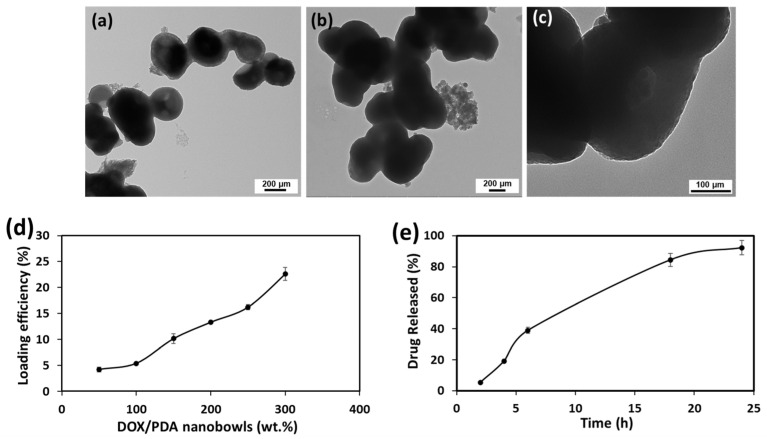
Transmission electron microscope (TEM) images of polydopamine mesoporous nanobowls coated with gadolinium (con. of gadolinium_100 µg/mL), before (**a**) and after (**b**) loading with doxorubicin, respectively, and (**c**) is the high-magnified view of image (**b**). Figure (**d**) shows the loading efficiency of DOX in gadolinium-coated PDA mesoporous nanobowls (con. of gadolinium_100 µg/mL) and figure (**e**) is the release of DOX from gadolinium-coated PDA mesoporous nanobowls (con. of gadolinium_100 µg/mL) in pH 5.6.

PDA nanobowls are rich in active functional groups and aromatic rings, which allow them to load various types of anti-cancer drugs including doxorubicin (DOX) [37]. Herein, using aromatic rings of PDA, the DOX molecules attached to the gadolinium-coated PDA nanobowls by π–π stacking interactions [30,37]. The TEM images in Figure 6a,b show that the thickness of gadolinium-coated PDA nanobowls increased significantly after loading with DOX. The high-magnified view in Figure 6c suggests the deposition of DOX on the surface of the nanobowls, and the insides of the cavity and mesochannels are also filled with DOX molecules. The distinctive peak at around 590 nm confirms the adsorption of DOX into the nanosystem. These samples were examined after loading the DOX and washing out the unbound DOX from the suspension (Figure S3). As observed, in comparison to the gadolinium-coated PDA nanobowls, the dox concentration in the supernatant decreased significantly in every wash, and after the fourth wash, DOX was removed completely from the nanosystem (Figure S3). A higher loading efficiency was observed with the non-coated PDA nanobowls compared to the gadolinium-coated one (Figure 6d) [30]; it is assumed that the space of the nanobowls is partially preoccupied due to the coating of gadolinium. However, the loading efficiency is still higher compared to nonporous PDA nanobowls, which indicates a higher capacity for loading drugs into the PDA nanobowls, confirming the strong contribution of electrostatic attractions resulting from the addition of π–π stacking into the drug loading process due to their negative surface charges. It is assumed that considerably larger available spaces, such as mesopores and cavities, allow greater drug adsorption into the nanosystem even after the coating of gadolinium. This is an advantage of the PRIMERS with respect to drug loading.

Previous studies have reported that acidic environments are generally ideal conditions to mimic the tumor cell extracellular microenvironment [37]. In our study, drug release profiles were characterized in an acidic environment by considering previous investigations. In addition to doxorubicin, other anti-cancer drugs such as paclitaxel (PTX) also show a similar drug release pattern (Figure S4), where higher release is observed in acidic environments like DOX. Previous studies demonstrate that due to the protonation of the amine group of PDA in an acidic environment, *π*–*π* stacking between PDA and anti-cancer drug molecules is disrupted [30,37]. In terms of the therapeutic efficacy of drug-loaded PDA nanobowls, a significant increase in cancer cell death was observed in a previous study in DOX-loaded PDA nanobowls compared with DOX alone [30]. Additionally, PDA nano bowls have been found to be efficient not only in loading various other anti-cancer drugs, but also in killing other types of cancer cells. As demonstrated in Figure S5, paclitaxel (PTX)-loaded PDA nanobowls efficiently killed lung cancer cells (A549 cells) and, more importantly, showed a continuous release of both drugs for more than 48 h.

#### 3.2.3. PRIMERS Loading AntiCD40 into Gadolinium-Coated PDA Nanobowls and In Vitro Release Profile

Polydopamine is considered a biomimetic analogue of natural photoprotective materials, and has the capacity to adsorb and covalently interact with proteins via supramolecular interactions, including electrostatic, van der Waals, or hydrogen bonds [43,44].

**Figure 7:**
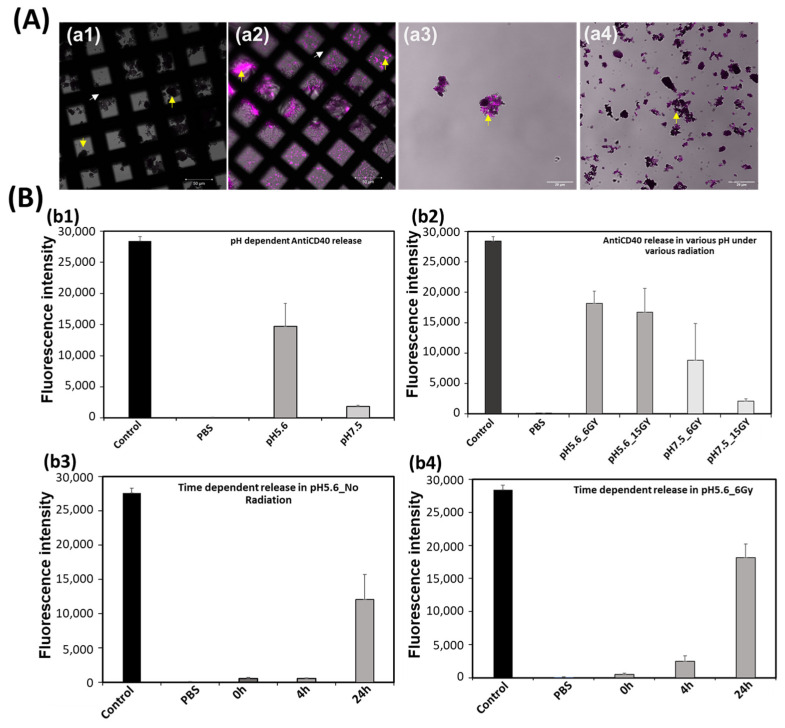
Figure (**A**) confocal microscopy images, herein, (**a1**) is PDA nanobowls only, (**a2**) is fluorophore-tagged antiCD40 only, (**a3**,**a4**) are antiCD40 (fluorophore tagged) loaded PDA nanobowls, with and without coated with gadolinium respectively; herein, the white arrow pointing the TEM grid in the image (**a1**,**a2**), while the yellow arrow (**a1**–**a4**) pointing the sample. Figure (**B**) is the fluorescence intensity profile of antiCD40 (Alexa fluor 700) release from PDA mesoporous nanobowls in various pH environments; herein, for (**b1**,**b2**) the incubation time was 24 h only, and for (**b3**,**b4**) there are various incubation duration as it is time-point study (0, 4, and 24 h).

Additionally, it has been demonstrated that covalent bioconjugation to proteins could proceed when the catechol groups are oxidized to quinones that are susceptible to nucleophilic attack by amines and thiols through Michael addition and Schiff-based reactions [44,45]. Inspired by the protein adsorption efficiency of PDA [46], in our study, for the first time, Alexa fluor 700-tagged antiCD40 was loaded into the gadolinium-coated PDA nanobowls. It is demonstrated that the sustained presence of agonistic antiCD40 within the tumor microenvironment provides significant support to continue the activity of infiltrating CD40+ APCs (antigen-presenting cells) in boosting tumor-specific cytotoxic T cell action, in particular for poorly immunogenic tumors in various cancerous tumors, including prostate and pancreatic tumors [13,47]. Thus, antiCD40 therapy demonstrates a tumor growth-inhibitory effect, providing potent apoptotic signals to the carcinoma cells (such as prostate, pancreatic, and cervical cancer) that likely stimulate the immune system, resulting in the abscopal effect [13,16,48]. The confocal microscopy images in Figure 7A confirm the successful loading of fluorophore-tagged antiCD40 into the PDA nanobowls with gadolinium coating (Figure 7A(a2,a3) (Figure 7A(a3)) is high magnified view of Figure 7A(a2))) and into non-coated ones (Figure 7(a4)). Herein, Figure 7A(a1) is PDA nanobowls only. After several washes with MES buffer, no fluorophore was observed in the supernatant, confirming the stability of the antiCD40 in the PDA nanobowls. Later on, the in vitro release profile of antiCD40 was investigated in various environments with and without exposure to radiation (Figure 7B). Herein, an acidic environment has been found most favorable in inducing the release of antiCD40. As observed, at pH 5.6, a significant amount of antiCD40 is released in 24 h (Figure 7B(b1)). It is assumed that the bond between PDA and antiCD40 is disrupted in an acidic environment, causing the release of antiCD40. Lower pH is favorable for the protonation of the amine group of PDA, which tends to disrupt the bonding between PDA and molecules [30,37]. Remarkably, the results show higher antiCD40 release under radiation (Figure 7B(b2)). Moreover, a faster release was observed under radiation compared to samples without any radiation at 4 h (Figure 7B(b3,b4)), indicating the strong contribution of radiation in activating the release of antiCD40. However, no noticeable impact was observed with various doses of radiation in this release profile. These results suggest that a certain pH environment is crucial in releasing antiCD40, and radiation is beneficial to inducing faster release in an acidic environment.

## 4. Conclusions

In summary, we have introduced PRIMERS—PDA mesoporous nanobowls loaded with immunoadjuvants with the potential to serve as fiducial markers for combination therapy, with image contrast that could allow for image-guided drug release and monitoring. The use of PRIMERS is intended as an alternative to the currently used fiducial markers, proffering multi-functionality that can be leveraged for image-guided radioimmunotherapy, with the precise local delivery of drugs that can be modulated by external stimulus. This study provides a valuable reference for further work toward developing and optimizing biocompatible and multifunctional fiducials that can significantly potentiate treatment efficacy during radio-immunotherapy, and minimize collateral damages or toxicities.

## Data Availability

Data are contained within the article or Supplementary Materials. Data are also available on request from the corresponding author.

## Supplementary Materials

The following supporting information can be downloaded at: https://www.mdpi.com/article/10.3390/pharmaceutics16111481/s1, Table S1: Table showing the dynamic light scattering and zeta potential measurement of the PDA nanobowls after coating with various concentrations of gadolinium; Figure S1: Showing the graphical representation of (A) average particle size and (B) zeta potential of the synthesized gadolinium coated PDA nanobowls, (C) is demonstrating the size analysis of the PDA nanobowls, herein, the data was generated by analysis hundreds of nanoparticles from TEM image, and three different TEM images of each sample were analyzed, and (D) FTIR spectra of PDA nanobowls and gadolinium coated PDA nanobowls; Figure S2: Confocal microscopy images of TC-1 cells, showing cellular uptake of polydopamine (PDA) nanobowls (at around 200 nm sized) after 24 h of incubation., a_1_ and a_2_ are the bright field image and merge image of only cells respectively, and b_1_ and b_2_ are the bright field image and merge of the cells that incubated with PDA nanobowls respectively. Herein, cells were stained with DAPI (blue) and the red signal is coming from the fluorophore dye (rhodamine 6 g) attached to the PDA nanobowls, and the green is the cell membrane dye (Alexa Fluor 488 WGA); Figure S3: (A) Showing the distinctive DOX peak at around 590 nm confirms adsorption of DOX into the nanobowls, and (B) Standard curve of DOX; Figure S4: Showing the cumulative PTX release in various pH; Figure S5: Showing the efficiency of PTX loaded PDA nanobowls in killing cancer cells (A549 cells), compared with the free PTX (left: 48 h and right: 72 h).
