# Supplementary material for: PRIMERS: Polydopamine Radioimmunotherapy with Image-Guided Monitoring and Enhanced Release System
Source: Pharmaceutics. 2024 Nov 20;16(11):1481. doi: 10.3390/pharmaceutics16111481 (PMC11597857; doi:10.3390/pharmaceutics16111481)
Supplement: Supplementary file 1 [file pharmaceutics-16-01481-s001.zip › pharmaceutics-3306684-supplementary.pdf]

Supporting Information

PRIMERS: Polydopamine Radioimmunotherapy with Image-guided Monitoring and Enhanced Release System

Shahinur Acter [1,*], Lindokuhle M. Ngema[1,3], Michele Moreau[1], Debarghya China[4], Akila Viswanathan[1], Kai Ding[1], Yahya E. Choonara[3], Sayeda Yasmin-Karim[2], and Wilfred Ngwa[1,*]

Sample ID	Diameter/Size (nm)	Polydispersity Index (PDI)	Zeta Potential (mV)
PDA nanobowls_ Gd con.1	243.3 ± 7.41	0.42 ± 0.025	-6.88 ± 0.87
PDA nanobowls_Gd con. 2	142.9 ± 8.22	0.33 ± 0.071	-4.48 ± 0.13

Table S1. Table showing the dynamic light scattering and zeta potential measurement of the PDA nanobowls after coating with various concentrations of gadolinium.

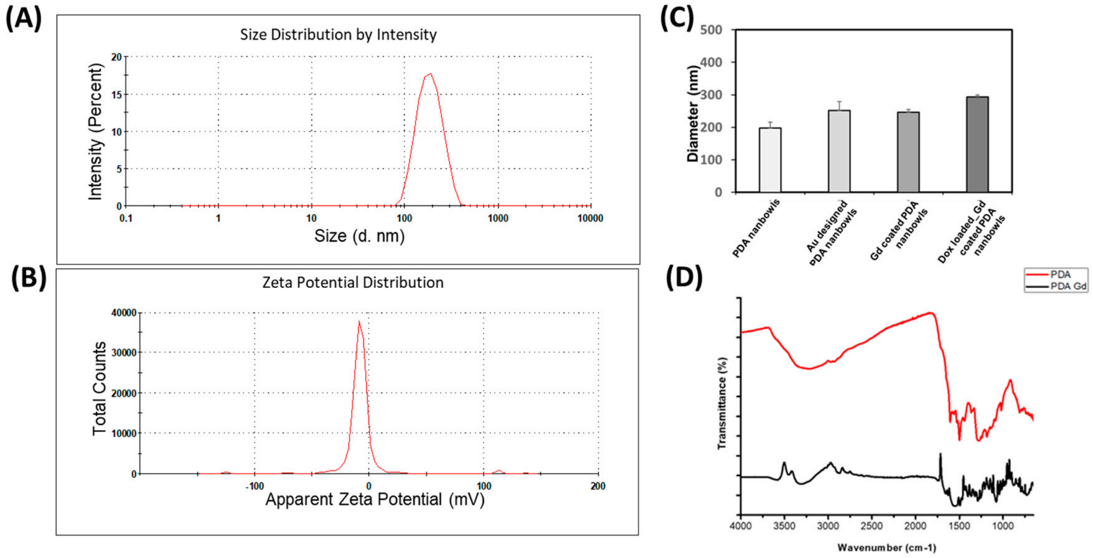

Figure S1. Showing the graphical representation of (A) average particle size and (B) zeta potential of the synthesized gadolinium coated PDA nanobowls, (C) is demonstrating the size analysis of the PDA nanobowls, herein, the data was generated by analysis hundreds of nanoparticles from TEM image, and three different TEM images of each sample were analyzed, and (D) FTIR spectra of PDA nanobowls and gadolinium coated PDA nanobowls.

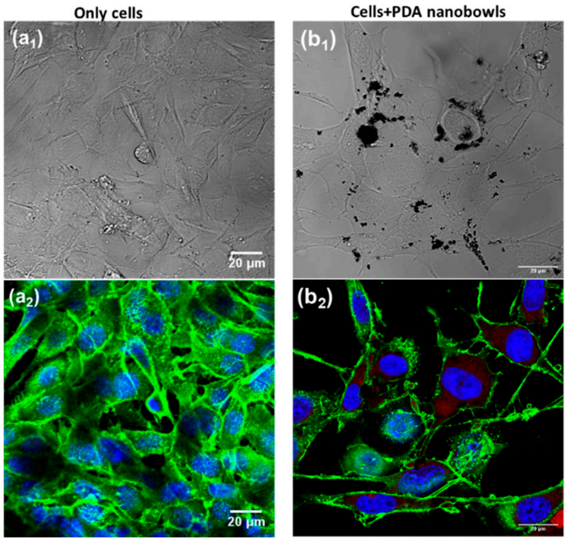

Figure S2. Confocal microscopy images of TC-1 cells, showing cellular uptake of polydopamine (PDA) nanobowls (at around 200 nm sized) after 24h of incubation., a_1 and a_2 are the bright field image and merge image of only cells respectively, and b_1 and b_2 are the bright field image and merge of the cells that incubated with PDA nanobowls respectively. Herein, cells were stained with DAPI (blue) and the red signal is coming from the fluorophore dye (rhodamine 6g) attached to the PDA nanobowls, and the green is the cell membrane dye (Alexa Fluor 488 WGA.).

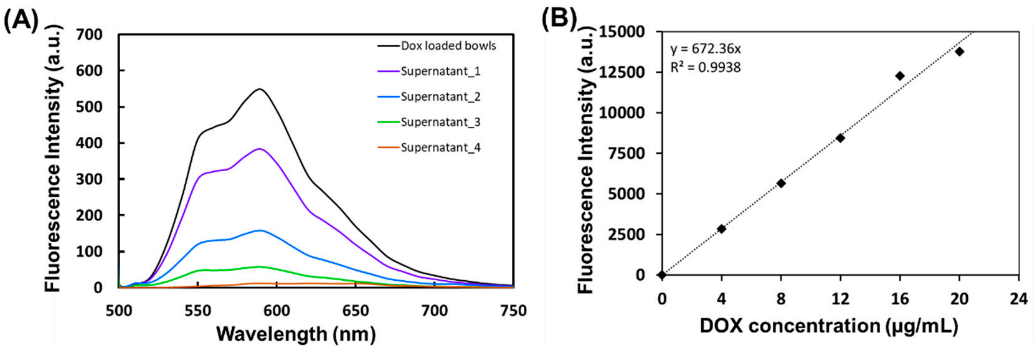

Figure S3. (A) Showing the distinctive DOX peak at around 590 nm confirms adsorption of DOX into the nanobowls, and (B) Standard curve of DOX

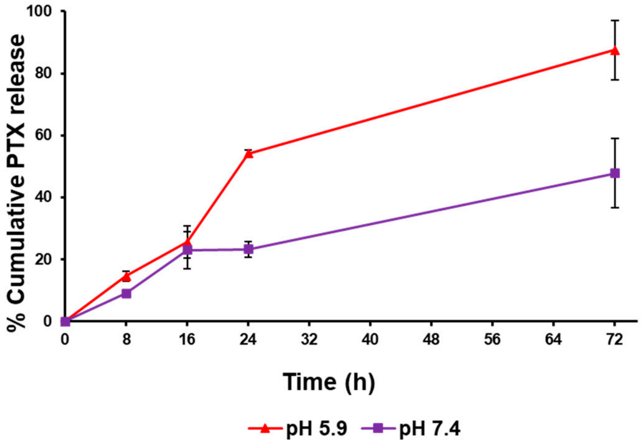

Figure S4. Showing the cumulative PTX release in various pH.

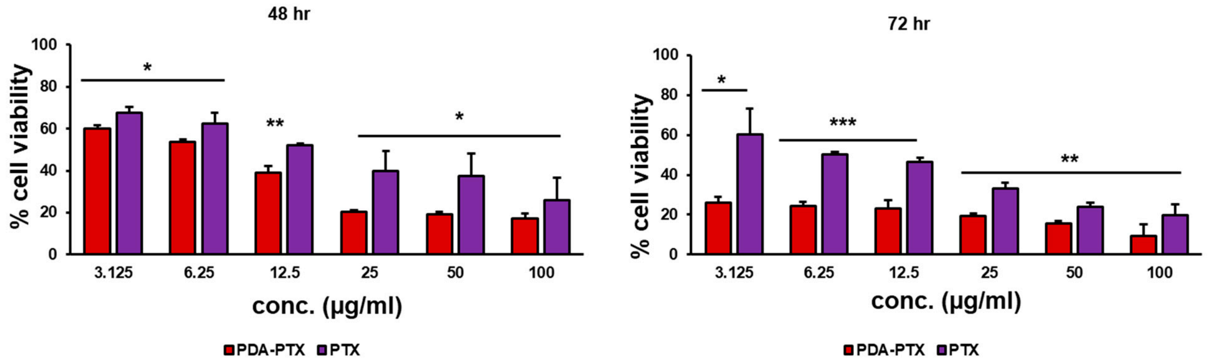

Figure S5. Showing the efficiency of PTX loaded PDA nanobowls in killing cancer cells (A549 cells), compared with the free PTX (left: 48 hrs and right: 72 hrs).
